# Scattering Elimination in 2D IR Immune from Detector Artifacts

**DOI:** 10.1021/acs.jpcb.4c04220

**Published:** 2024-08-27

**Authors:** Anneka Miller Casas, Nehal S. Idris, Victor Wen, Joseph P. Patterson, Nien-Hui Ge

**Affiliations:** Department of Chemistry, University of California, Irvine, California 92697-2025, United States

## Abstract

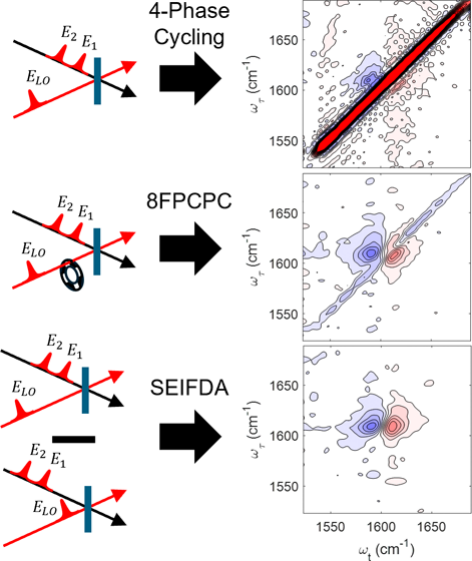

Highly scattering samples, such as polymer droplets or solid-state powders, are difficult to study via coherent two-dimensional infrared (2D IR) spectroscopy. Previously, researchers have employed (quasi-) phase cycling, local-oscillator chopping, and polarization control to reduce scattering, but the latter method poses a limit on polarization-dependent measurements. Here, we present a method for Scattering Elimination Immune from Detector Artifacts (SEIFDA) in pump–probe 2D IR experiments. Our method extends the negative probe delay method of removing scattering from pump–probe spectroscopy to 2D experiments. SEIFDA works well for all polarizations when combined with the optimized noise reduction scheme to remove additive and multiplicative noise. We demonstrate that our method can be employed with any polarization scheme and reliably lowers the scattering at parallel polarization to comparable levels to the conventional 8-frame phase cycling with probe chopping (8FPCPC) at perpendicular polarization. Our system can acquire artifact free spectra in parallel polarization when the signal intensity is as little as 5% of the intensity of the interference between the pump pulses scattered into the detector. It reduces the time required to characterize the scattering term by at least 50% over 8FPCPC. Through detailed analysis of detector nonlinearity, we show that the performance of 8FPCPC can be improved by incorporating nonlinear correction factors, but it is still worse than that of SEIFDA. Application of SEIFDA to study the encapsulation of Nile red in polymer droplets demonstrates that this method will be very useful for probing highly scattering systems.

## Introduction

Two-dimensional infrared (2D IR) spectroscopy is a well-established method for determining many useful aspects of molecular systems including time-resolved dynamics and molecular structure.^1,2^ Many of these experiments require spectra acquired with different polarization schemes in order to scale different Feynman pathways and extract information such as the relative angles of the transition dipoles.^3−7^ Often, samples of interest such as metal–organic frameworks,^8,9^ porous silica,^10−13^ fibrils,^14^ zeolite,^15^ minerals,^16^ and pelleted samples^17^ cause significant scattering. In the box-cars geometry, researchers have used quasi-phase cycling^13,18^ or a combination of choppers and shutters.^19^ In the pump–probe geometry, researchers have used 4-phase cycling,^20^ population-time modulation,^21^ nearly crossed polarization,^7^ and a combination of probe chopping and polarization control to remove the scattered light.^8,22^ More recently, researchers have employed a strong probe, weak pump combination as well as polarization control.^17^ Polarization control limits the experiments available and therefore what can be determined about the system of interest. Here we present a method for scattering removal which can be employed with any polarization scheme and reliably lowers the scattering to comparable levels to the chopper and polarization control method introduced by Biaz et al.^22^

In heterodyne detected third order spectroscopy, when the probe serves as the local oscillator, the total intensity on the detector is expressed as eq 1,^8^
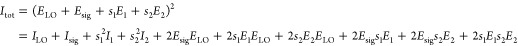
1We will use *E* for electric field terms and *I* for intensity terms. *E*_LO_ and *I*_LO_ are the local oscillator electric field and intensity, respectively. *E*_sig_ and *I*_sig_ are the electric field and intensity of the signal, respectively. *E*_1_ and *E*_2_ are the pump electric fields. *I*_1_ and *I*_2_ are the pump intensities. Finally, *s*_1_ and *s*_2_ are scattering constants for these pump fields, respectively. In our setup, we utilize a pulse shaper to generate two collinear pump fields.^20,23^ It is well established that a 4-phase cycling scheme removes all heterodyned scattering terms.^4,20,24^ Rotating the phase of the first pump pulse (first term in parentheses) and the second pump pulse (second term) according to eq 2
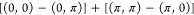
2removes all the intensity terms (*I*_LO_, *I*_sig_, *s*_1_^2^*I*_1_, *s*_2_^2^*I*_2_) including the strong local oscillator background while also removing the heterodyne detected scattering terms (*s*_1_*E*_1_*E*_LO_, *s*_2_*E*_2_*E*_LO_) without chopping either the pump or the probe.^8^ This means that every shot contributes to the *E*_sig_*E*_LO_ term. However, this scheme does not remove *s*_1_*s*_2_*E*_1_*E*_2_. Therefore, after combining *I*_tot_ from 4 shots based on phase cycling in eq 2, and neglecting the small signal terms (*s*_1_*E*_1_*E*_sig_, *s*_2_*E*_2_*E*_sig_) and the factor of 2, we are left with *S*_tot_:
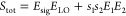
3When the *s*_1_*s*_2_*E*_1_*E*_2_ term is much smaller than *E*_LO_, this term can be neglected as demonstrated by Donaldson et al.^17^ When a sample causes significant scattering and the phase and amplitude of *E*_LO_ cannot be independently controlled, *s*_1_*s*_2_*E*_1_*E*_2_ can become dominant. This is especially true when the pump is significantly stronger than the probe, as is typically the case to improve the signal-to-noise ratio (SNR). Because the phase of *E*_LO_ cannot be independently controlled, the *s*_1_*s*_2_*E*_1_*E*_2_ scattering term has the same sign as *E*_sig_*E*_LO_, and cannot be removed using phase cycling. This is what motivated researchers to employ the 8-frame phase cycling with probe chopping (8FPCPC)^8,22^ with two sets of the 4-phase cycling in eq 2 applied to the pump pulses. The probe is present in the first 4 frames and the probe is chopped in the second 4 frames
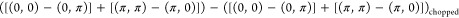
4Taking the difference between the two sets gives the signal *S*_8FPCPC_:
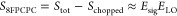
5Here *S*_chopped_ is obtained after 4-phase cycling of the detector intensity when the probe beam is chopped, *I*_chopped_:
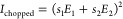
6If the detector were perfectly linear, this scheme would, on average, result in the isolation of *E*_sig_*E*_LO_, without the need for perpendicular polarization to suppress scattering.

In this paper, we will demonstrate why the 8FPCPC cannot effectively isolate *E*_sig_*E*_LO_ for all polarizations when using a HgCdTe (MCT) detector. Furthermore, we will present a new method for Scattering Elimination Immune from Detector Artifacts (SEIFDA), as expressed in eq 7,
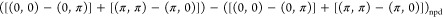
7where npd indicates negative probe delay. Taking the difference between the two sets results in
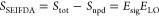
8where *S*_npd_ is the signal obtained after applying 4-phase cycling to the detector intensity when there is a negative probe delay with respect to the pump beam, *I*_npd_:
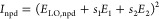
9Here the subscript npd indicates that the probe arrives at least 10 ps before the pump arrives at the sample. We will show that when the detector nonlinearity and multiplicative (convolutional) noise are correctly accounted for, eq 8 is truly equal to *E*_sig_*E*_LO_. SEIFDA is a 2D analog of the pump–probe scattering elimination method which also utilizes a negative delay between the pump and probe.^10^

We first demonstrate the effectiveness of the SEIFDA using a sample which does not have any intrinsic scattering to verify that no artifacts are introduced and quantify the residual scattering. Next, we use it to study a highly scattering sample containing nonionic block copolymer coacervates.^25^ We confirm the encapsulation of Nile red within an amphiphilic block copolymer polyethylene glycol-*block*-polycaprolactone (PEG_45_-*b*-PCL_30_). In order to compare the effectiveness of the new scattering removal method to the 8FPCPC commonly used,^8,9,14,16,22^ we characterize the scattering reduction using a 100-μm pinhole in place of a sample. This results in generation of scattering terms only, allowing us to accurately quantify the remaining scattering. Finally, we demonstrate that SEIFDA characterizes the scattering term at least 50% faster than the 8FPCPC and discuss some additional considerations that researchers may need when designing scattering removal in heterodyne detected experiments.

## Methods

### Samples

To validate the effectiveness of this method, we characterized the remaining scattering in a sample without intrinsic scattering, a sample of *N*-*tert*-butyl-2,2-dimethyl-propionamide in D_2_O. In this sample, there was both a large scratch and pieces of dust on the window. The concentration was 45 mM. The thickness was 100 μm. FTIR confirmed that the OD was approximately 0.1.

Additionally, we looked at Nile red, a hydrophobic dye commonly used as a model system for encapsulation,^26^ within polyethylene glycol-*block*-polycaprolactone (PEG_45_-*b*-PCL_30_).^27^ To prepare the samples, 1.12 mM of the polymer PEG_45_-*b*-PCL_30_ solution was prepared by dissolving the polymer in a 5 mM Nile red solution in dioxane. By volume, 70% of this mixture was transferred to a 1.5 mL Eppendorf tube, and 30% D_2_O was added to reach 3.5 mM Nile red and 0.84 mM polymer. For the samples of Nile red without polymer, a solution of 3.5 mM Nile red in 70% by volume dioxane and 30% D_2_O was prepared. The solutions were vortexed for approximately 25 s before they were pipetted into a sample cell. Optical and confocal laser scanning microscopy was used to determine the size of the polymer droplets. The diameters of the droplets were between 10 and 20 μm.

### Spectroscopy

We acquired all the linear IR spectra using a Jasco 4700 FTIR purged with dry air. Harrick cells with 100-μm Teflon spacers and CaF_2_ windows were used. For the Nile red samples, the solvent peaks (70% by volume dioxane and 30% D_2_O) were removed by subtracting the solvent spectrum acquired immediately before using a thickness-matched spacer and cell.

We acquired 2D IR data in the pump–probe geometry using a setup previously described.^28^ The time zero was found using a position on the sample cell with significant scattering. We scanned the coherence time between the first and second pump pulse, τ, from 0 to 4.5 ps in 0.025 ps steps using an AOM-based pulse shaper. The pump–probe delay time was set by a computer-controlled translation stage. The probe spectrum was calibrated using water lines. The pump spectrum was calibrated using neat acetone and a 100-μm thick sample of 5 mM benzanilide dissolved in dimethyl sulfoxide. The probe frequency, ω_t_ is directly reported by the spectrometer. The pump frequency, ω_τ_ is obtained by Fourier transforming the time domain data along τ. For the 2D IR spectra presented in this paper, the waiting time between the pump and probe, *T*_w_, was set to 0.3 ps. When used, the chopper was placed immediately before the focusing parabola and synchronized to the laser. The pump spectrum was monitored to confirm that it was not clipped by the chopper. When both ⟨YYZZ⟩ and ⟨ZZZZ⟩ were collected, at the sample, the probe was polarized at 45° to the table. After the sample, prior to detection, the ⟨YYZZ⟩ and ⟨ZZZZ⟩ were separated using an analyzer which allowed the ⟨ZZZZ⟩ to pass and reflected the ⟨YYZZ⟩. The ⟨YYZZ⟩ signal was then transmitted through another polarizer to clean the signal. When only ⟨ZZZZ⟩ spectra were collected, the λ/2 waveplate prior to the signal generation was used to change the probe polarization to match the pump. The MCT we use operates as photoconductive, as most MCTs have been constructed since at least the 1970s.^29^ It has 2 rows with 32 pixels in each row which allows us to detect ⟨YYZZ⟩ and ⟨ZZZZ⟩ simultaneously, when desired. Optimized referencing^30,31^ was implemented using a separate MCT reference array detector. Every 40 shots (4 phases × 10 steps along τ) we collect 2 blank shots (shots which do not include the pump), unless we are chopping. When chopping, because our chopper was operating at 0.5 kHz and we were utilizing a 1-kHz laser, we had to collect twice the number of blank shots to obtain the same number of shots which contained the *E*_LO_.

### Computational

For Nile red, anharmonic DFT calculations were performed using the Gaussian 16 package^32^ with functional B3LYP and basis 6-311g++(d,p) with an implicit water solvent.

## Results and Discussion

### Effectiveness of Scattering Removal Using SEIFDA

**Figure 1:**
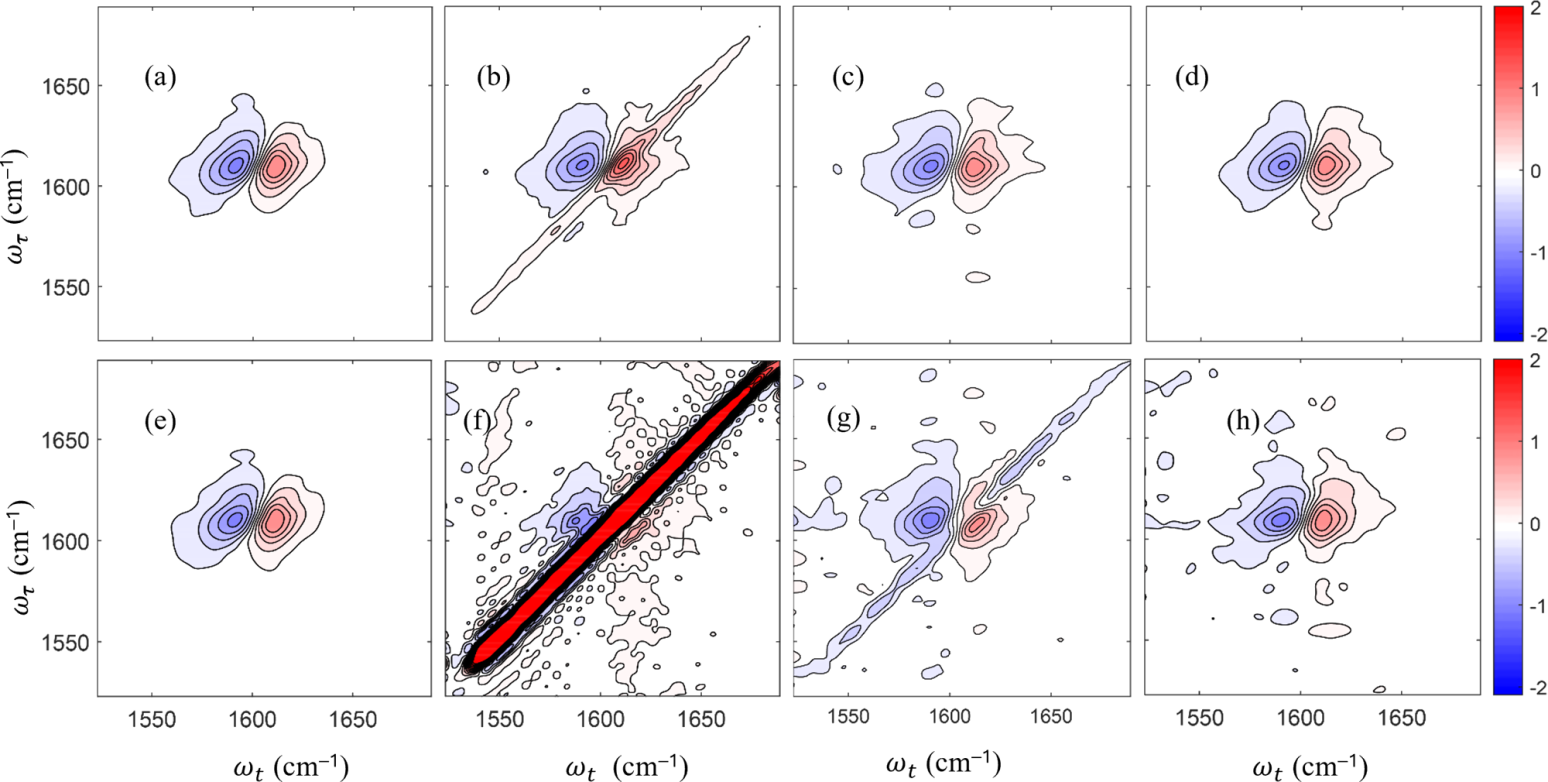
Efficacy of SEIFDA compared to 8FPCPC on a sample of *N*-*tert*-butyl-2,2-dimethyl-propionamide in D_2_O. Parallel polarization ⟨ZZZZ⟩ was used. Data were normalized based on the maximum value after scattering was removed. Waiting time was 0.3 ps. The top row, (a–d), were acquired using the low gain setting of the detector. The bottom row, (e–h), were acquired using the high gain setting. The first column, (a) and (e), were acquired in the same position with no observed scattering using the 4-phase cycling scheme. (b) and (f) were acquired in two different positions with moderate and significant scattering, respectively, using the 4-phase cycling scheme. The scattering is approximately 38% of the signal intensity in (b), and 750% in (f). Columns (c) and (g), are the result after removing the scattering using 8FPCPC. The remaining scattering in (c) is 17% of that in (b); and in (g) it is 9.1% of that in (f). (d) and (h) are the results after applying SEIFDA. The remaining scattering in (d) is 5.6% of that in (b); and in (h) it is 0.85% of that in (f).

The largest issue with scattering removal methods that utilize *E*_LO_ chopping^8,19,22^ is that MCTs are more linear for very low levels of light and become less linear with higher levels of light, essentially reaching saturation slowly.^29,33^ In MCTs, as photon irradiance increases, the number of excess carriers affects the total carrier density and carrier lifetime.^29,34,35^ In heterodyne detected spectroscopy, the most intense field on the detector is *E*_LO_. Therefore, this field contributes the most to how nonlinear the detector response will be. When determining how detrimental detector nonlinearity is to a measurement, we need to consider the difference in detector response across pixels and on individual pixels across shots. For signal intensities on the mOD level or lower, the difference in MCT reading across pixels is likely to be small, assuming the experiment is conducted within the full width half-maximum of the excitation source. The difference in MCT reading from shot to shot on a single pixel should be even smaller. When chopping the probe beam, however, the difference in MCT readings between chopped and unchopped shots can be exceptionally large in comparison to the dynamic range of the detector. Because MCTs tend to be more linear for very low intensities of light and have a smaller response with higher levels of light,^29^ as discussed later and demonstrated in Figure 3, the *s*_1_*s*_2_*E*_1_*E*_2_ scattering term will appear to be much larger when measured with *E*_LO_ chopped (equal to *S*_chopped_) compared to when measured with *E*_LO_ unchopped (a component in *S*_tot_). Therefore, eq 4 will result in residual negative *s*_1_*s*_2_*E*_1_*E*_2_ along the diagonal, as seen in Figure 1g, rendering scattering removal by 8FPCPC ineffective. This is why previous researchers had to use the ⟨YYZZ⟩ polarization control as well as 8FPCPC^8,14^ (although in the case of 2D IR microscopy,^22^ this was also to separate the collinear pump and probe).

In order to fully remove the *s*_1_*s*_2_*E*_1_*E*_2_ scattering term, we need to acquire a spectrum of *s*_1_*s*_2_*E*_1_*E*_2_ independently of *E*_sig_*E*_LO_, then we can calculate *E*_sig_*E*_LO_ + *s*_1_*s*_2_*E*_1_*E*_2_ – *s*_1_*s*_2_*E*_1_*E*_2_. To collect this term in a manner that is unaffected by detector nonlinearity, we devised the SEIFDA method where the probe can be moved enough in time before the pump so that the sample will have no memory of the probe when the pump arrives, eliminating the four-wave mixing *E*_sig_ terms, allowing us to isolate the scattering term *s*_1_*s*_2_*E*_1_*E*_2_. The intensity on the detector with a sufficiently negative probe delay, eq 9, can be reduced to
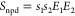
10after 4-phase cycling is applied. Subtracting *S*_npd_ from *S*_tot_ allows us to obtain the heterodyned signal *E*_sig_*E*_LO_ we are interested in. Furthermore, because *I*_npd_ is measured in the presence of *E*_LO_, both *S*_tot_ and *S*_npd_ are collected in the same detector linearity regime. As a result, the spectrum is immune to detector nonlinearity artifacts. We will discuss this aspect more in the section on the detector nonlinearity.

For our MCT detector, most of the pixel response has decayed after about 2000 ns, although 5000 ns are required for it to fully decay. If the probe arrives within tens of ps of the scattering, the pixel response will not have appreciably changed. We confirmed this was true for our detector for up to 30 ps. When measuring different material systems in general, researchers need to make sure that this negative delay time is much longer than the dephasing time of vibrational coherence generated by the probe pulse. They should also confirm that the detector response does not appreciably decay during the required time for the coherence to fully decay. This value will vary from one detector to another as it depends on the pixel response time. Most of the oscillators we are interested in will have no memory of the probe if it arrives 10–30 ps before the pump. In principle, it is also possible to choose a positive probe delay instead. However, the required positive delay time would need to be sufficiently longer than the decay time of population dynamics or thermal effects generated by the pump pulses, which can be much longer than the coherence decay time and thus not as practical.

Because 2D spectra, as well as pump–probe spectra, and the scattering term *s*_1_*s*_2_*E*_1_*E*_2_, depend on the intensity of the pump, which fluctuates over time, one potential issue with the SEIFDA method is that the long-term fluctuations in pump intensity may play a larger role here than the 8FPCPC method because the time between taking *S*_tot_ and *S*_npd_ is typically longer than the time between taking *S*_tot_ and *S*_chopped_. In practice, we found that the fluctuations in the pump are well accounted for using the terms used to characterize the multiplicative noise.^30,31^ We will discuss in more detail how multiplicative noise is treated and additional considerations in a later section.

To validate the effectiveness of SEIFDA and to compare it with 8FPCPC, we characterized the remaining scattering in a sample without intrinsic scattering. Figure 1 shows the results when we applied both methods of scattering removal to a sample of *N*-*tert*-butyl-2,2-dimethyl-propionamide in D_2_O. In this sample, there was both a large scratch and pieces of dust on the window. We chose two sample locations to demonstrate two cases: moderate and significant scattering where the scattering intensity is about 38 and 750% of the signal intensity in Figure 1b,f, respectively, estimated based on the maximum noise intensity in *S*_npd_ and the ground state bleach signal in *S*_SEIFDA_. The top (bottom) row spectra were obtained when the detector was set to low (high) gain. Figure 1a,d are taken in the same position with no observed scattering using the 4-phase cycling scheme as a standard for comparison.

Because the 4-phase cycling scheme eliminates all heterodyne detected terms but does not impact the *s*_1_*s*_2_*E*_1_*E*_2_ term, comparing the on-diagonal absolute value intensity before and after removing scattering using the SEIFDA method allows us to estimate the percentage of *s*_1_*s*_2_*E*_1_*E*_2_ removed with respect to the 4-phase cycling scheme. We examined the diagonal in the region from 1643 to 1679 cm^–1^ to estimate scattering. This region is spectrally clear without features from the sample. In Figure 1c, the ground state bleach is obviously distorted compared to Figure 1a by residual scattering left after 8FPCPC (17% of scattering in Figure 1b). In Figure 1g, we can see the significant residual scattering along the diagonal after 8FPCPC (9.1% of scattering in Figure 1f). Furthermore, the sign is flipped in Figure 1g compared to Figure 1f because the *s*_1_*s*_2_*E*_1_*E*_2_ term acquired without *E*_LO_ is greater than when acquired with *E*_LO_, as expected if detector nonlinearity is the explanation for imperfect scattering removal when relying on probe chopping. In contrast, in Figure 1d,h, SEIFDA reduced the remaining *s*_1_*s*_2_*E*_1_*E*_2_ to 5.6 and 0.85% of the intensity remaining after 4-phase cycling for the low gain and high gain cases shown here, respectively. Clearly, SEIFDA suppresses scattering much more effectively than 8FPCPC. For both methods, scattering removal is more effective at the high gain setting than the low gain setting, but SNR is lower at the high gain setting. We will further discuss the significance of the low gain and high gain settings later in the paper.

### Application to Nile Red Encapsulation

**Figure 2:**
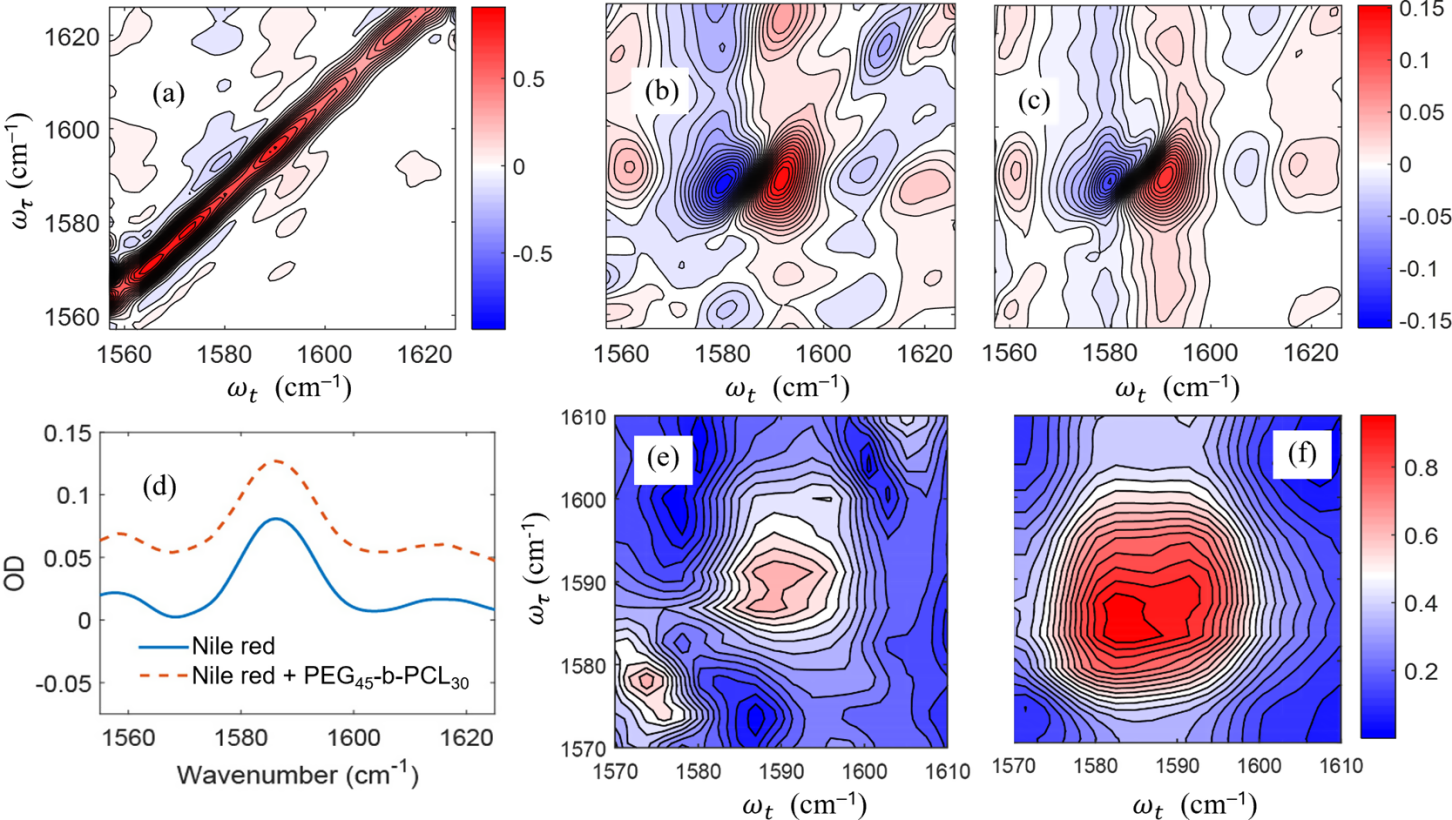
(a) and (b) Absorptive 2D IR spectra of Nile red with PEG_45_-*b*-PCL_30_ in dioxane-water solution taken by the 4-phase cycling scheme and SEIFDA, respectively. High gain setting used. (c) Absorptive 2D IR spectrum of Nile red with solvent only. Low gain setting and 4-phase cycling scheme used. Parallel polarization used for all 2D IR spectra. Waiting time 0.3 ps. (d) FTIR of Nile red in solution with and without PEG_45_-*b*-PCL_30_. (b) and (c) were plotted with the same number of contours and same minimum and maximum. (e) and (f) Absolute value nonrephasing spectra of Nile Red with and without polymer, respectively. (e) and (f) were normalized and plotted with the same number of contours as each other.

We applied the SEIFDA method to confirm the encapsulation of small molecule Nile red, a hydrophobic dye commonly used as a model system for encapsulation,^26^ within amphiphilic block copolymer PEG_45_-*b*-PCL_30_. Figure 2 shows the 2D IR and FTIR results for a Nile red vibrational mode with and without PEG_45_-*b*-PCL_30_. DFT calculations indicate that this mode has contributions from the C=O stretching and ring breathing. Harmonic DFT calculations put this mode at 1609 cm^–1^ and anharmonic DFT calculations put it at 1576 cm^–1^. In the FTIR, this peak is centered at 1586 cm^–1^. We attribute some of the discrepancy between the DFT calculations and the reality to the difference in solvent. For the DFT calculations, we used an implicit water solvent rather than a more accurate, but significantly more computationally expensive, explicit mixture of 70% by volume dioxane and 30% water.

The differences between Nile red in solution without and with PEG_45_-*b*-PCL_30_ are subtle in the FTIR spectra, Figure 2d. Similarly, after scattering removal from the spectra acquired with polymer, the differences in the 2D IR absorptive spectra are also very small between Figure 2b,c. However, when we extract the nonrephasing spectrum using a Hilbert transform,^24,36^ we see that there are two overlapping peaks^37,38^ for the Nile red with PEG_45_-*b*-PCL_30_. We have assigned the higher frequency peak to the free Nile red because it is the closest in frequency to what we observe for the Nile red in solvent only and the lower frequency to the encapsulated Nile red. The intensities of the two peaks are nearly equal, consistent with the estimation that ∼50% of the Nile red in the focus is encapsulated.

### Detector Nonlinearity

Because we think that the detector nonlinearity^29,39^ is most likely the cause of the ineffective scattering removal if the 8FPCPC were employed without polarization control, we needed to accurately estimate the detector nonlinearity. To do so, we characterized the detector response with the laser intensity on the detector significantly attenuated with neutral density (ND) filters and then again with fewer ND filters.^40,41^ We used the pump pulse shaper to continuously adjust the laser intensity.

**Figure 3:**
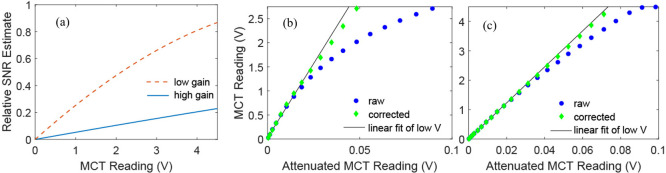
(a) Estimate of relative SNR as a function of MCT readings at the low gain (dashed) and high gain (solid) detector settings. Estimate is based on rational function fit of detector readout for the 16th pixel. (b) and (c) Detector response for the low and high gain settings used in this paper, respectively, for the 16th pixel. The raw is the detector readout whereas the “corrected” response was calculated using eqs 11, 14, and 15. The line is a linear fit to the low voltage range.

The detector nonlinearity depends on the gain setting used. Often, the most linear gain setting does not have the best SNR.^30^ Estimates for the SNR^30,31,35^ for our detector are shown in Figure 3a. In this paper, we will compare two gain settings, as previewed in Figure 1. The first is the most linear, the highest gain setting. However, increasing the gain increases the dark noise, so this gain setting does not achieve the maximum SNR for our detector.^30^ We will refer to this setting as high gain for the rest of the paper. The second setting used is a lower gain setting that does achieve the maximum SNR. Of the settings that reach the maximum SNR, this setting has the largest “dynamic range.” For this paper, we will define the top of the “dynamic range” to be a deviation from linearity by approximately 10%.^33^ We will refer to the second setting as low gain for the rest of the paper. Note that we utilized a different low gain setting in this paper from the one utilized by Feng et al.^30^

Figure 3b,c show the MCT detector raw response measured at the low and high gain settings, respectively. The exact top of the dynamic range for the raw response differs from pixel to pixel, however, we estimate it to be around 3 V for the high gain and around 1.25 V for the low gain. For the high gain setting, most pixels deviate from linearity by less than 1% when the MCT reading is below approximately 1 V. For the low gain setting, there is no range where the majority of pixels deviate from linearity by less than 1%. Most pixels deviate by less than 5% from the predicted linear response below a reading of 1 V for the low gain setting.

To reduce the effects of detector nonlinearity on spectra, one can attempt to correct detector response. Detector nonlinearity has long been characterized and corrected using polynomial fits.^40−43^ It has been shown that, when the nonlinearity is small, the detector response can be corrected using eq 11,^41^
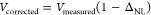
11where *V*_corrected_ and *V*_measured_ are the corrected and measured detector response in volts and Δ_NL_ is the detector nonlinearity correction factor, which can be calculated according to the polynomial in eq 12,^41^
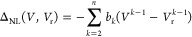
12where *V*_r_ is a reference voltage. The coefficients, *b*_*k*_ , are determined by fitting the data according to eq 13(41)
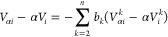
13The factor α is the transmission through the ND filters, *V*_*i*_ is the MCT reading of the *i*^th^ measurement, and *V*_α*i*_ is the corresponding attenuated MCT reading in volts. In the saturation regime, Δ_NL_ is negative, so (1 – Δ_NL_) > 1. We used *n* = 3 as this produced more consistent results.^41^ Because *V*_α*i*_ and *V*_*i*_ are not collected simultaneously, there may be some variability in the laser intensity. In theory, this could be corrected by treating α as a variable rather than a constant, but in practice this introduces more noise into the Δ_NL_.^41^

The Δ_NL_(*V*, *V*_r_) depend significantly on the *V*_r_ used. When we applied the nonlinear correction factors to real 2D IR data, we found that the *V*_r_ value that best removed scattering produced artifacts in the spectra when we used eqs 12 and 13 as written. In order to find the Δ_NL_ that gives the best agreement for the 2D IR signal with the linear MCT response (for the high gain setting, this occurs at the range of mean *I*_LO_ below 0.78 V where the observed response is very linear), we came up with a new expression to calculate *b*_*k*_ , using
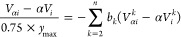
14then replace the fixed *V*_r_ in eq 12 with a moving reference *V*_*i*_ as shown in eq 15,
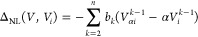
15where *y*_max_ is the maximum value of (*V*_α*i*_ – α*V*_*i*_) that occurs at the saturation limit. Although applying the nonlinearity correction factors can extend the dynamic range, data still needs to be acquired far enough from the saturation value that there is a consistent, observable difference in reading between shots. We selected 75% of the maximum of the differences as the approximate top of the range where there are still reliable changes in the detector response with changing signals. Eq 15 accounts for the nonlinearity contributed by the detector and preamplifier at the same time.^34^ The corrected responses shown in Figure 3b,c are quite close to the linear fit through the low-voltage data points.

**Figure 4:**
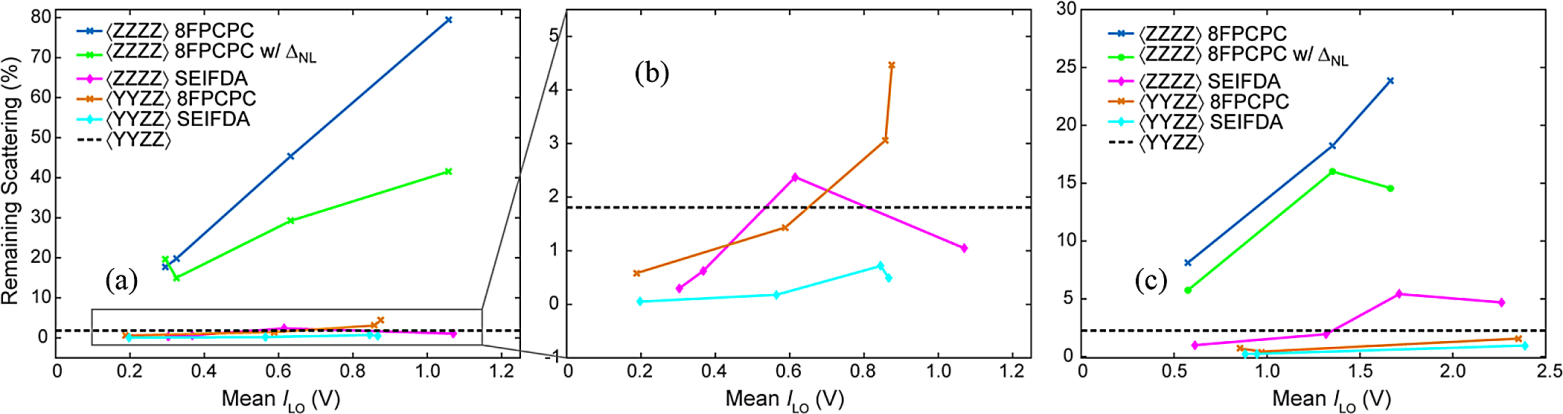
To estimate the scattering reduction as a function of the local oscillator intensity, we acquired scattering-only spectra using a 100-μm pinhole to scatter pump into the probe path for both the (a) low gain and (c) high gain detector settings. To better visualize the low gain results, panel (b) zooms in on the bottom region of (a). Scattering reduction was estimated by comparing the on-diagonal maximum after applying a specific method to the scattering detected for the parallel polarization ⟨ZZZZ⟩ at the same time. The average scattering reduction achieved through 4-phase cycling by changing the polarization from parallel to perpendicular ⟨YYZZ⟩ is shown in a dashed black line. The Δ_NL_ factors were calculated using eqs 14 and 15.

To better remove scattering using 8FPCPC, one can determine nonlinearity correction factors for each pixel at the relevant voltages used in the experiment, and then apply the nonlinearity correction factors to the data. In practice, this approach does not sufficiently remove scattering, as shown in Figure 4. Because 8FPCPC requires data to be collected across nearly the full detector dynamic range, the exact pixel nonlinearity becomes very important. It is difficult to make the Δ_NL_ work well for the whole dynamic range. As we will discuss later, we do not apply nonlinear correction to every shot, but to the averaged data. Furthermore, small changes in the orientation of the cable connecting the detector to the preamplifier change the dark noise slightly. These make determining the exact pixel nonlinearity challenging. We believe that this is why the implementation of 8FPCPC for ⟨ZZZZ⟩ does not work well even with Δ_NL_.

As far as we can tell, determining Δ_NL_ using eqs 14 and 15 is a novel method for calculating the corrected detector response. For the low gain setting, using eq 14 we can extend the dynamic range to about 2 V. This range was determined by comparing slices of 2D IR spectra of *N*-*tert*-butyl-2,2-dimethyl-propionamide in D_2_O at different gain settings to ensure that the line shape was not visibly distorted. By extending the dynamic range from ∼1.25 to ∼2 V, the SNR is increased by 46%. Therefore, using eq 14 to correct data acquired over the top of the detector dynamic range enables researchers to acquire data with a higher SNR.

### How Does Scattering Removal Using SEIFDA Compare to Other Methods?

The effectiveness of the different methods for scattering removal depends on the intensity of the local oscillator, because it is the greatest contribution to the detector nonlinearity caused by a change in the density of charge carriers and because it is a source of noise, even after shot-to-shot noise is reduced by referencing.^30,31^Figure 4 compares the effectiveness of the different scattering removal methods at different mean local oscillator intensities on the detector. To estimate the scattering removal, we used a pinhole to scatter the pump into the probe path. All methods are compared using the scattering in 4-phase cycling with parallel polarization as a reference. Because the polarizer extinction coefficient is very nearly constant throughout the intensities measured here, for clarity, we indicate the remaining scattering of 4-phase cycling with perpendicular polarization by a dashed straight line. Across the dynamic range, SEIFDA brought the remaining scattering to a comparable level with the ⟨YYZZ⟩ 8FPCPC, for both the parallel and perpendicular polarizations. Furthermore, when operating near the top of the dynamic range, the 8FPCPC with ⟨YYZZ⟩ performed worse than 4-phase cycling with polarization control alone for the low gain setting used, as seen in Figure 4b. This behavior shows that the effect of detector nonlinearity can overwhelm the ability of polarization control to suppress scattering.

As the local oscillator intensity increases, the noise also increases. After referencing, the effect is small, however, we believe that this accounts for the upward trend in remaining scattering for all methods as the mean *I*_LO_ increases. Using eqs 14 and 15 to correct the data with Δ_NL_, the remaining scattering can be lowered for the methods which use probe chopping, however, there is still significant scattering. For very strongly scattering samples, if a researcher wishes to collect spectra using parallel polarization, they should consider the relative intensity of the probe to the pump^17^ then utilize ND filters to the keep the overall intensity on the detector within the very linear regime of their detector, as was required for Figure 1h, when the scattering was very intense in comparison to the signal. In our system, we have gone further than Figure 1h and achieved 0.3% remaining scattering with the mean of *I*_LO_ was set to 0.3 V (data not shown). This means that we can use SEIFDA to acquire artifact free spectra in parallel polarization when the signal intensity in *S*_SEIFDA_ is as little as 5% of the scattering intensity in *S*_npd_.

Researchers who use 2D MCT focal plane arrays^44^ may spread their signal over more pixels resulting in a more linear MCT response. This approach will improve scattering removal, but may reduce SNR depending on their dark noise levels.

### Considerations on Noise Suppression and Data Acquisition Efficiency

When we compare these methods of scattering removal, they have different time and laser drift considerations. The 8FPCPC method may be implemented by alternating consecutive shots, as we did, or by grouping chopped and unchopped shots for measuring *S*_tot_ and *S*_chopped_ as shown in eq 4. The laser repetition rate and the time required for the detector response to decay should be considered when determining how to implement either the 8FPCPC or our SEIFDA.

While considering laser drift, we should determine the proper order for data acquisition. In general, it is best to acquire data for each different pump phase combination at a single τ then move to the next τ until all τ’s have been collected, then begin repeats. This is because if the pump and probe are not perfectly synchronized in time, there may be a very small drift in their timing. This ordering is important for all terms that contain *E*_LO_ (*E*_sig_*E*_LO_, *s*_1_*E*_1_*E*_LO_, *s*_2_*E*_2_*E*_LO_), however, it is unimportant for terms created by collinear excitation sources that contain only interference between these pulses (in the pump–probe geometry, *s*_1_*s*_2_*E*_1_*E*_2_).

The data presented in this paper applied the optimized noise suppression method invented by Feng et al.^30,31^ that was initially derived to eliminate noise based on two consecutive shots^30^ and later generalized to scenarios with complex chopping or phase cycling patterns.^31^ If one chops the probe beam in every other shot in order to eliminate the scattering terms, one needs to then compare the noise of every other shot. Making this adjustment to the referencing scheme is straightforward, using the generalized Δ operator, Δ_1–3_ , in reference (31). To build a matrix of blank shots collected on the signal and reference detectors with a set number of shots with the local oscillator present, one now needs to collect twice as many blank shots as before because half of the shots collected will not have any light because the probe is chopped. This increases the amount of time required to eliminate the scattering using 8FPCPC, but only marginally. We typically spend about 5% of data acquisition time on collecting blank shots.^31^ This was increased to 10% when acquiring data using 8FPCPC.

Because using SEIFDA to measure *I*_npd_ requires moving the delay stage, it would take significant experimental time if one were to move the stage for every other shot or after every coherence time τ (this would be after 4 shots for 4-phase cycling). It is more efficient to collect a complete spectrum of *S*_tot_ followed by a complete spectrum of *S*_npd_. This increases the time between collecting the terms. It takes approximately 1 min to collect a complete spectrum in our current setup (this corresponds to 151 steps in τ × 4 phases × 99 internal repeats). The effect of long-term laser drift can be compensated for using the multiplicative (convolutional) noise correction term.^30,31^ Referencing is vital to the application of SEIFDA. The remaining scattering in Figure 1d goes from 5.6%, with referencing applied, to 58% without referencing. For Figure 1h, it goes from 0.86% when referencing is applied, to 17% without referencing.

The multiplicative noise has been treated by considering an *F* factor that depends on the heterodyne detection technique and experimental details.^30,31^ For a 2-phase cycling scheme that flips the pump phase between 0 and π, *F* = *I*_LO_^*^I_Pu_^*^ + *I*_LO_^′^*I*_Pu_^′^ where *I*_LO_ and *I*_Pu_ are the intensity of the probe and pump beams, respectively; * and ′ refer to the shots where the pump phase is 0 and π, respectively.^31^ In principle, we should use the true intensities of relevant beams,^30^ and factor out *F* on a shot-to-shot basis. In practice, we can only use the detector outputs that contain shot noise and detector noise, and factoring out *F* using averaged intensities has been shown to be sufficient.^30^ Therefore, we treat the fluctuations of LO and pump separately on an average basis.

The intensity of fluctuating LO, *I*_FLO_, can be well approximated by summing *I*_tot_ over 4-phase cycling,
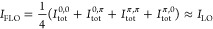
16because *E*_sig_*E*_LO_ and *s*_1_*s*_2_*E*_1_*E*_2_ , terms with opposite pump phases (shown in the superscripts), cancel each other. To monitor *I*_Pu_ , one can acquire a pump spectrum during every 2D IR spectrum acquisition. In our current setup, this method is feasible if we use one array for pump monitoring and another array for data collection. However, this method does not completely remove the dependence on the pump because it is the intensity at the sample, not at the detector that matters, so fluctuations in the pump focus are not accounted for. If the pump and probe are generated by the same source, collecting a pump spectrum is unnecessary as the pump and probe intensity fluctuations are strongly correlated. Therefore, we can use the local oscillator intensity to adjust for the laser drift in the pump as well. Because we shape the pump in the frequency domain, the individual colors may fluctuate slightly differently between the pump and the probe. We assume that *I*_Pu_(*t*, λ) = *I*_LO_(*t*) × *c*(λ) and the pump probe focus overlap at the sample is nearly constant in time. We monitored pump and probe spectra overnight, and confirmed that these assumptions held for the 12 h monitored. We spectrally average *I*_FLO_ over pixels to give *I*_FPu_ to account for the intensity of fluctuating pump.

When using SEIFDA to isolate *E*_sig_, we calculate *E*_sig_ according to eq 17 to express it in absorbance change in e-base,
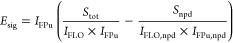
17where the subscript npd indicates that these terms are taken at a negative probe delay. For *I*_FLO_, and *I*_FLO,npd_ , there is a unique term for each τ. For *I*_FPu_ and *I*_FPu,npd_ , it is averaged over the τ scan. We obtained a slightly higher SNR and better scattering removal when the data were averaged first, then additive noise was removed followed finally by removal of multiplicative noise.

When using 8FPCPC to isolate *E*_sig_ , there is no good way to estimate *F* when the probe beam is chopped. Because the chopped shots typically appear either every other shot or every 4 shots, the effect of long-term laser drift is expected to be less than SEIFDA. The simplest solution which gave us the best scattering removal was to simply exclude the chopped shots from the calculation of multiplicative noise for the data included in this paper and use eq 18 to calculate *E*_sig_ ,
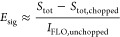
18where *I*_FLO,unchopped_ is found similar to eq 16 where the phases are summed over for unchopped shots. When Δ_NL_ detector nonlinear correction factors were applied, the Δ_NL_ used for each pixel was determined based on the average probe blank shot intensity during the run on that pixel. These factors could instead be calculated for each shot, however, when this was attempted, data acquisition was slowed because too much memory was required (this was true whether the calculation was done during or after data acquisition because either way, we need to save significantly more data). *E*_sig_ was calculated with individual pixel’s Δ_NL_ according to eq 19
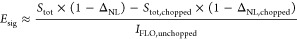
19When calculating Δ_NL_ using eqs 14 and 15, it is often not necessary to “correct” the chopped shots because the intensity on the detector is typically at the very linear range and thus (1 – Δ_NL,chopped_) ≈ 1.

Removing the scattering using SEIFDA applied as a full, well averaged spectrum for *S*_tot_ followed by a well averaged spectrum for *S*_npd_ only works if the scattering material is not moving. If the scattering material is moving in and out of the pump–probe overlap region in the sample on a fast time scale compared to the time it takes to acquire a spectrum, then we must reduce the time to acquire a spectrum. We collect spectra by acquiring each of the 4-phases at τ_1_, then move to τ_2_ and cycle through the 4-phases, then repeat until we get to τ_max_. We repeat this process approximately 100 times then save the data. This was chosen to minimize the time where no data is collected. If the scattering material moves on the minute scale, but does not appreciably move within ∼1200 ms, the approximate minimum time to acquire a spectrum of *S*_tot_ and another of *S*_npd_ for a 1 kHz laser, then we could set the internal repeats to 0. If the scattering moves faster than this, we can split the acquisition of τ’s such that we acquire a portion of the τ’s for *S*_tot_ followed by acquisition of those same τ’s for *S*_npd_ followed by the next set of τ’s for *S*_tot_ and repeat until the full spectra are acquired. This was not required for any of the samples we looked at, but we imagine it may be necessary for samples with microscopic bubbles and solvents with very low viscosity.

**Figure 5:**
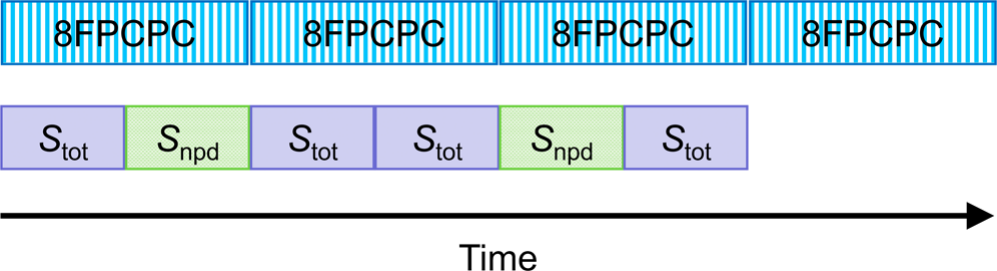
Comparison of data acquisition for 4 averages (or waiting times) of 8FPCPC (top row) and 4 averages (or waiting times) of SEIFDA (bottom row). In the top row, the blue vertical lines represent chopped shots whereas the white vertical lines represent unchopped shots. In the bottom row, the purple and green blocks represent the time periods that are used to collect *S*_tot_ and *S*_npd_ spectra, respectively. One half of the shots in the 8FPCPC row will not contain signal. In contrast, one-third of the shots in SEIFDA will not contain signal. For SEIFDA, the number of shots without signal can be reduced further, depending on the laser stability.

If only one average and one waiting time is required, the 8FPCPC and the SEIFDA methods require the same number of shots. However, when acquiring data with different waiting times for extracting the frequency-frequency correlation function, we found that SEIFDA with referencing^30,31^ enabled us to reduce the frequency we had to acquire scattering-only spectra. We can arrange to start and end with *S*_tot_, acquire *S*_npd_ followed by two more *S*_tot_ at the desired waiting times in between every acquisition of *S*_npd_, then utilize the same *S*_npd_ to remove scattering from the *S*_tot_ acquired before *S*_npd_ and the *S*_tot_ acquired after *S*_npd_, as shown in Figure 5. We used this approach to reduce the scattering-only shots by least 50% when compared to the 8FPCPC. The frequency needed to collect scattering-only spectra can be reduced further depending on the stability of the light source used.

When the multiplicative noise is removed following our discussion above, researchers utilizing the box-cars geometry could also reduce the frequency they acquire scattering-only terms. Consider a combination of choppers and shutters,^19^ instead of interspersing the chopped and shuttered shots, they could group the shuttered and unshuttered shots to collect separate spectra. They could then use the same spectrum of scattering only to remove the scattering from multiple spectra with signal and scattering. One remaining issue with this method would be the phasing issues which may occur. Before implementing this method, researchers would have to evaluate how frequently they need to adjust the phase of the data.

Here we would like to make a quick note on referencing and scattering removal. Because we split the probe and the reference early in our setup,^28^ before the sample, and utilize separate detectors for signal detection and reference detection, our reference is inherently scattering free. This makes it much simpler to implement both referencing and SEIFDA. If this is not the case, for example, if researchers use one row of a dual-stripe MCT array to detect the reference and the other row to detect the signal, or edge pixels on the signal detector to serve as reference pixels,^45^ or several rows in a 2D MCT focal plane array,^44^ there may be pump scattering in the reference. In this situation, we recommend utilizing crossed polarization for the reference, to reduce scatter onto the reference detector. As discussed previously,^31^ to ensure that referencing does not incorrectly add a background to the real signal, the reference detection must satisfy the condition that ⟨Δ*I*_ref_⟩ = 0, and hence the reference cannot contain any pump-induced signal, including scattering.

When polarization control is insufficient to remove scattering from the reference, then special attention should be paid to the implementation of the ***B*** matrix. The ***B*** matrix must be calculated using truly blank shots, shots collected with all electric fields besides the local oscillator blocked. SEIFDA should be implemented using the same uncontaminated ***B*** for *S*_tot_ and *S*_npd_ when the scattering detected on the reference detector, *q*_1_*q*_2_*E*_1_*E*_2_ is not negligible. Referencing will result in adding (−*q*_1_*q*_2_*E*_1_*E*_2_***B***) to *S*_tot_ and *S*_npd_ , but calculating (*S*_tot_ – *S*_npd_) will remove the reference contamination. This explains how SEIFDA coupled with optimized referencing^30,31^ is expected to remove all scattering terms even when the reference is contaminated by scattering.

Additive noise is largely introduced by the local oscillator, so reducing *E*_LO_ on the detector can reduce additive noise.^36^ However, the referencing scheme reduces additive noise to nearly the noise floor.^30,31^ This changes the SNR considerations when determining how intense the *E*_LO_ should be. Although the spectrum is independent of the *E*_LO_, because the term we are interested in detecting, *E*_sig_*E*_LO_, scales with the intensity of the *E*_LO_, increasing the local oscillator intensity improves SNR. Additionally, increasing the *E*_LO_ relative to the pump, reduces the effects of scattering on the spectrum.^17^ When doing single color 2D IR experiments using only one OPA, we cannot independently vary the relative intensity of *E*_LO_. However, this relationship between *E*_LO_ and pump intensity is still useful to keep in mind for experimental configurations that allow independent adjustment of *E*_LO_. In this case, increasing *E*_LO_ in conjunction with the optimized referencing scheme^30,31^ can increase SNR and enhance scattering removal.

## Conclusions

In conclusion, we have developed a method of scattering removal, SEIFDA, which works well for all polarization combinations. Furthermore, it requires at least 50% less time to characterize the scattering than the more commonly used 8FPCPC method. SEIFDA avoids the artifacts caused by detector nonlinearity by acquiring all data points in the same detector linearity regime. SEIFDA is made possible through the reduction of both additive and multiplicative noise with optimized referencing. We have demonstrated the usefulness of SEIFDA on determining the encapsulation of small molecule Nile red in PEG_45_-*b*-PCL_30._ Furthermore, we have presented a method for correcting the detector nonlinearity which will enable researchers to acquire higher SNR data. In the future, researchers will be able to use SEIFDA to obtain spatial and dynamic information for samples with low signal to scattering ratios as SEIFDA provides improved flexibility in polarization control, detector dynamic range, and a reduction in the time required to characterize scattering, enabling researchers to acquire more averages.
